# Uptake and yield of HIV testing and counselling among children and adolescents in sub-Saharan Africa: a systematic review

**DOI:** 10.7448/IAS.18.1.20182

**Published:** 2015-10-14

**Authors:** Darshini Govindasamy, Rashida A Ferrand, Stephanie MS Wilmore, Nathan Ford, Saeed Ahmed, Hoviyeh Afnan-Holmes, Katharina Kranzer

**Affiliations:** 1 Health Systems Research Unit, South African Medical Research Council, Cape Town, South Africa; 2 Department of Clinical Research, London School of Hygiene and Tropical Medicine, London, United Kingdom; 3 Biomedical Research and Training Institute, Harare, Zimbabwe; 4 Centre for Clinical Microbiology, University College London, London, United Kingdom; 5 HIV/AIDS Department, World Health Organization, Geneva, Switzerland; 6 Tingathe Outreach Program, Baylor College of Medicine Children's Foundation Malawi, Lilongwe, Malawi; 7 Department of Medicine, Baylor College of Medicine, Houston, TX, USA

**Keywords:** adolescents, children, HIV testing and counselling, sub-Saharan Africa

## Abstract

**Introduction:**

In recent years children and adolescents have emerged as a priority for HIV prevention and care services. We conducted a systematic review to investigate the acceptability, yield and prevalence of HIV testing and counselling (HTC) strategies in children and adolescents (5 to 19 years) in sub-Saharan Africa.

**Methods:**

An electronic search was conducted in MEDLINE, EMBASE, Global Health and conference abstract databases. Studies reporting on HTC acceptability, yield and prevalence and published between January 2004 and September 2014 were included. Pooled proportions for these three outcomes were estimated using a random effects model. A quality assessment was conducted on included studies.

**Results and discussion:**

A total of 16,380 potential citations were identified, of which 21 studies (23 entries) were included. Most studies were conducted in Kenya (*n*=5) and Uganda (*n*=5) and judged to provide moderate (*n*=15) to low quality (*n*=7) evidence, with data not disaggregated by age. Seven studies reported on provider-initiated testing and counselling (PITC), with the remainder reporting on family-centred (*n*=5), home-based (*n*=5), outreach (*n*=5) and school-linked HTC among primary schoolchildren (*n*=1). PITC among inpatients had the highest acceptability (86.3%; 95% confidence interval [CI]: 65.5 to 100%), yield (12.2%; 95% CI: 6.1 to 18.3%) and prevalence (15.4%; 95% CI: 5.0 to 25.7%). Family-centred HTC had lower acceptance compared to home-based HTC (51.7%; 95% CI: 10.4 to 92.9% vs. 84.9%; 95% CI: 74.4 to 95.4%) yet higher prevalence (8.4%; 95% CI: 3.4 to 13.5% vs. 3.0%; 95% CI: 1.0 to 4.9%). School-linked HTC showed poor acceptance and low prevalence.

**Conclusions:**

While PITC may have high test acceptability priority should be given to evaluating strategies beyond healthcare settings (e.g. home-based HTC among families) to identify individuals earlier in their disease progression. Data on linkage to care and cost-effectiveness of HTC strategies are needed to strengthen policies.

## Introduction

In recent years children and adolescents have emerged as a priority group for HIV prevention and care services. The prevalence of undiagnosed HIV infection is substantially higher in children and adolescents compared to adults and, among those diagnosed HIV positive and eligible for treatment, coverage of antiretroviral therapy (ART) is low (34%) [1]. HIV testing and counselling (HTC) is the critical first step to accessing HIV treatment and prevention services. In addition, risk behaviours are often developed during adolescence and HTC is an opportunity to engage this age group, including those who test HIV negative, to promote healthy sexual practices through counselling and linkage to other health services, such as circumcision and contraception. The well-described burden of survival to older childhood with untreated, vertically acquired HIV infection [2], the young age of sexual debut – up to a quarter of 15- to 19-year-olds in sub-Saharan Africa (SSA) report sex before the age of 15 – and the high HIV incidence rates in SSA further highlight the importance of effective and acceptable strategies for HTC in older children and adolescents in this region [3].

Recognizing the need to prioritize adolescents as a key population for HIV prevention, the World Health Organization (WHO) developed specific HTC guidelines for adolescents in 2013 [4]. Most of the WHO recommendations were based on very low quality evidence, and the guidelines highlighted the need to establish comparative effectiveness of interventions to improve access to HTC. Despite the lack of head-to-head comparisons of different HTC strategies among this age group, existing studies reporting on a specific strategy may provide insight into acceptability, yield and prevalence. We conducted a systematic review to investigate the acceptability, yield and prevalence of different HTC strategies in children and adolescents in SSA.

## Methods

### Search strategy and study inclusions

The search strategy aimed to identify evidence from randomized and non-randomized trials, prospective and retrospective cohort studies, cross-sectional studies and programme evaluations that reported on HTC among children and adolescents (5 to 19 years) with sufficient data to calculate acceptance and yield of HIV-positive diagnoses. Studies were omitted if participants did not receive their test results (i.e. anonymized surveys) or if the age range did not overlap with the targeted age range (5 to 19 years) by at least three years. Studies conducted in antenatal settings as part of the prevention of mother-to-child transmission (PMTCT) and in inpatient, outpatient, STI and TB clinics were only included for full text review if the abstract indicated that the age range overlapped with the targeted age range (5 to 19 years) by at least three years. The search was limited to studies conducted in SSA and published between 1 January 2004 and 30 September 2014, with no language restrictions. See protocol (Supplementary file 1) and PRISMA checklist attached (Supplementary file 2).

An electronic search was conducted on MEDLINE, EMBASE and Global Health using a compound search strategy (Supplementary file 3). A checklist of known studies was used to ensure that our search strategy captured all relevant studies. In addition, abstracts of all conferences of the International AIDS Society were screened from 2010 to 2013 to identify studies that may have been recently completed but not yet published. Reference lists of all eligible studies and systematic reviews were searched for additional articles, and authors of potentially eligible and included studies were contacted to provide age-stratified data. Two attempts were made to contact authors.

### Data extraction

All references identified by the compound search strategy were imported into EndNote, and titles and abstracts were screened independently by two investigators (KK, DG). Full texts of potential studies were then obtained and the inclusion criteria applied. Final study inclusion was based on consensus between investigators (DG, KK). Data was then entered from each selected study onto a standardized data extraction form (DG) and cross-checked (KK). The following variables were extracted: study design, study setting, HTC strategy, type of HIV screening test used, number of participants who were offered HTC, number of participants who accepted HTC and number of participants testing HIV positive.

### Definition of outcomes

The following definitions were applied: 1) *testing acceptance rate*, the proportion of individuals who underwent HTC and received their test results of those eligible for HTC; 2) *yield of new HIV-positive diagnoses*, the proportion of individuals who were newly diagnosed HIV positive of those who were eligible for HTC; and 3) *prevalence of new HIV-positive diagnoses*, the proportion of individuals who were newly diagnosed HIV positive of those who underwent HTC.

### Quality assessment

The quality of evidence among included published studies was assessed using standardized criteria which examined misclassification, selection and reporting bias by evaluating the following factors: description of HTC procedures, inclusion and exclusion criteria, sampling strategy, reporting of HTC outcome data, discussion of limitations and sub-group analyses performed. Study quality was classified accordingly: high (score 8 to 10), moderate (score 5 to 7) or low (score 4 to 0).

### Data analysis

For each included study, the numbers of individuals eligible, tested and tested positive were used to estimate proportions and corresponding 95% confidence intervals. Data were then pooled and stratified by testing strategy. On initial analysis, significant heterogeneity was found between studies. Therefore the pooled proportions of individuals accepting testing and testing positive (and 95% confidence interval, CI) were estimated with a random effects model, weighting for the inverse of the variance. Data analyses were conducted using Stata 12 (StataCorp, College Station, TX, USA).

## Results and discussion

**Figure 1:**
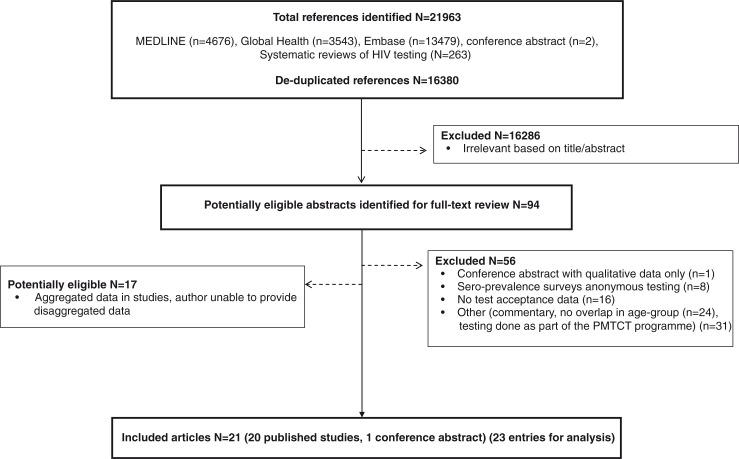
Selection process for the inclusion of studies.

A total of 16,380 deduplicated potentially relevant citations were identified, including three systematic reviews on HTC [5–7] that provided 263 references to be screened. Ninety-four abstracts were identified for full-text review (Figure 1). A total of 21 studies were potentially eligible as there was overlap in the age range, but the data presented in the publication were not sufficiently stratified by age to enable data extraction for the age group of interest. In addition, adolescents aged 15 to 19 were mainly grouped with the 20- to 24-year-olds. Authors of all of these studies were contacted, resulting in an additional four studies (five entries for analysis) being included in the review [8–11].

### Modes of HIV testing

The 21 studies (23 entries for analysis) included in the review (Table 1) reported data across eight countries: Kenya (*n*=5) [12–16], Uganda (*n*=5) [11, 17–19], Zimbabwe (*n*=4) [20–23], South Africa (*n*=3) [8, 24, 25], Tanzania (*n*=3) [9, 10], Malawi (*n*=1) [26], Sudan (*n*=1) [27] and Zambia (*n*=1) [28] (Table 1). Seven studies employed provider-initiated testing and counselling (PITC) for either inpatient (*n*=3) or outpatient (*n*=4) settings. Six studies were conducted in the context of sero-prevalence surveys, of which two provided HTC in the home environment and four used a mobile or outreach approach. A further four studies reported data from mass testing campaigns using outreach or home-based strategies. A family-centred approach was used in five studies, whereby patients known to HIV services were asked to identify other members in their households at risk of HIV infection. Finally, one study reported results from a school-linked testing campaign among primary schoolchildren aged 5 to 11 years [23]. Test uptake was comparable in girls and boys except for one study conducted in an outpatient setting in South Africa [25]. Fourteen studies reported HIV prevalence stratified by gender; of those, six [17, 19–22, 25] did not find any difference, and the remaining eight [8–10, 12, 14, 24, 26] found a higher prevalence in girls compared to boys. The majority of studies (*n*=22) used rapid point-of-care (POC) testing; testing conducted before 2005 was performed in the laboratory and results were either returned to participants at their homes or participants were asked to return to local primary healthcare clinics (PHC) for their test results. Most published studies on PITC, home-based HTC and outreach HTC were of moderate quality (*n*=15), with seven studies judged to be of low quality because of a high degree of selection bias due to the sampling strategy used (i.e. consecutive sampling) and HTC being performed at set times during the day (Supplementary file 4).

**Table 1:** Studies included in the review

Author, Year	Country, region	Year of the study	Setting	Context	HTC strategy	Testing method	Eligible age group	Median or mean age	Proportion female	Number offered testing	Number accepted testing	Acceptance rate			Total testing HIV positive	Yield^a^	Prevalence^b^			Quality of evidence
	
												Total	Female	Male			Total	Female	Male	
Ferrand *et al*. (2010)	Zimbabwe, Harare	2007 to 2008	Tertiary hospital, urban	Inpatient	PITC	POC testing	10 to 18 yrs	13 yrs IQR:11 to 16	0.43	215	197	91.6%	–	–	50	23.3%	25.4%	No significant association between sex and HIV status (*p*=0.25)		Moderate
Wanyenze *et al*. (2010)	Uganda, western	2005 to 2008	Tertiary hospital, urban	Inpatient	PITC	POC testing	15 to 17 yrs	–	0.47	148	141	95.3%	–	–	10	6.8%	7.1%	–	–	Moderate
Abbas *et al*. (2010)	Sudan, Khartoum	2007 to 2008	Tertiary hospital, urban	Inpatient	PITC	POC testing	1.5 mo to 14 yrs	5 yrs	0.42	127	106	83.5%	–	–	6	4.7%	5.7%	–	–	Low
Kankasa *et al*. (2009)	Zambia, Lusaka	2006 to 2007	Tertiary hospital, urban	Inpatient	PITC	POC testing	6 to 18 yrs	–	–	1785	1060	59.4%	–	–	248	13.9%	23.4%	–	–	Low
Ramirez-Avila *et al*. (2012)	South Africa, Durban (KwaZulu-Natal)	2008 to 2009	Secondary hospital, urban	Outpatient	PITC	POC testing	12 to 17 yrs	–	0.55	956	389	40.7%	49.0%	30.0% *p*<0.01	62	6.5%	15.9%	16.0%	16.0% *p*=0.99	Moderate
Kranzer *et al*. (2014)	Zimbabwe, Harare	2013	PHC	Outpatient	PITC	POC testing	6 to 15 yrs	9 yrs IQR: 7 to 11	0.47	2151	1534	71.3%	No significant association between sex and acceptance rate		82	3.8%	5.3%	No significant association between sex and HIV status		Moderate
Ferrand *et al*. (2010)	Zimbabwe, Harare	2009	PHC and ANC clinics, peri-urban	Outpatient	PITC (*n*=506), ANC (*n*=88)	POC testing	10 to 18 yrs	APC: 14 yrs ANC: 17 yrs	APC: 0.58 ANC: 1.00	594	573	96.5%	–	–	75	12.6%	13.1%	For APC, no significant association between sex and HIV status (*p*=0.35)		Moderate
Mongare *et al*. (2013)	Kenya	2009 to 2012	Primary healthcare facilities	Outpatient	Family-centred HTC	POC testing	0 to 15 yrs	–	–	22,688	7382	32.5%	–	–	839	3.7%	11.4%	–	–	N/A
Kulzer *et al*. (2012)	Kenya	2007 to 2009	Primary healthcare facilities	Outpatient	Family-centred HTC	POC testing	0 to 15 yrs	–	–	484	276	57.0%	–	–	50	10.3%	18.1%	–	–	Low
Were *et al*. (2006)	Uganda, districts of Tororo and Busia	2003 to 2004	Community, urban, rural	Home-based	Family-centred HTC	Laboratory testing, results provided at people's homes	6 to 10 yrs	–	0.48	604	602	99.6%	99.0%	99.0%	23	3.8%	3.8%	4.0%	4.0%	Low
Were *et al*. (2006)			11 to 17 yrs	–	0.48	737	734	99.6%	99.0%	99.0%	12	1.6%	1.6%	2.0%	2.0%	Low
Lugada *et al*. (2010)	Uganda, southeastern	2005 to 2007	Community, rural, urban	Home-based	Family-centred HTC	POC testing	6 to 14 yrs	–	0.53	1779	1055	59.3%	58.2%	60.6%	24	1.3%	2.3%	2.7%	1.8%	Moderate
Lugada *et al*. (2010)	Uganda, southeastern	2005 to 2007	PHC	Outpatient	Family-centred HTC	POC testing	6 to 14 yrs	–	0.53	979	96	9.8%	8.6%	11.1%	9	0.9%	9.4%	8.9%	9.8%	Moderate
Naik *et al*. (2012)	South Africa, Sisonke District (KwaZulu-Natal)	2009 to 2011	Community, rural	Testing campaign	Home-based HTC	POC testing	14 to 19 yrs	–	0.65	1011	867	85.8%	86.9%	85.6%	32	3.2%	3.7%	5.5%	0.3%	Moderate
Wachira *et al*. (2014)	Kenya, western	2009 to 2012	Community, urban/rural	Testing campaign	Home-based HTC	POC testing	13 to 18 yrs	15.3 yrs	0.50	34,607	34,410	99.4%	No significant association between sex and acceptance rate		162	0.5%	0.5%	Females had greater odds of testing HIV positive		Low
Vreeman *et al*. (2010)	Kenya, western	2008	Community, urban	Testing campaign	Home-based HTC	POC testing	18 mo to 13 yrs	–	0.48	2289	1294	56.5%	58.0%	55.2%	60	2.6%	4.6%	–	–	Moderate
Dalal *et al*. (2013)	Kenya, Lwak (Nyanza Province), Kibera (Nairobi)	2008	Community, urban/rural	Sero-prevalence survey	Home-based HTC	POC testing	<13 yrs	–	0.49	1234	1190	96.4%	96.8%	96.0%	136	11.0%	11.4%	12.9%	10.0%	Moderate
					13 to 17 yrs	–	0.51	3731	3236	86.7%	86.2%	87.3%	113	3.0%	3.5%	5.1%	1.82%	Moderate
Angotti *et al*. (2009)	Malawi, North, Central and South Regions	2004, 2006	Community, rural	Sero-prevalence survey	Home-based HTC	Laboratory testing, results provided at people's homes 2004, POC testing 2006	15 to 19 yrs	–	0.49	1076	1007	93.6%	93.5%	93.7%	6	0.6%	0.6%	1.0%	0.2%	Moderate
Kranzer *et al*. (2011)	South Africa, Cape Town	2010	Community, peri-urban	Sero-prevalence survey	Outreach (Mobile clinic with home-based invitation)	POC testing	15 to 19 yrs	–	–	140	119	85.0%	–	–	3	2.1%	2.5%	Females had greater odds of testing HIV positive		Moderate
Baisley *et al*. (2012)	Tanzania, Mwanza Region (Lake zone)	2007 to 2008	Community, urban, peri-urban	Sero-prevalence survey	Outreach HTC at central site (opt in)	POC testing	15 to 19 yrs	–	0.55	1302	786	60.4%	61.4%	59.1%	7	0.5%	0.9%	1.4%	0.3%	Moderate
Baisley *et al*. (2012)	Tanzania, Mwanza Region (Lake zone)	2007 to 2008	Community, urban, peri-urban	Sero-prevalence survey	Outreach HTC at central site (opt out)	POC testing	15 to 19 yrs	–	0.57	1223	1103	90.2%	91.1%	89.0%	11	0.9%	1.0%	1.6%	0.2%	Moderate
Isingo *et al*. (2012)	Tanzania, Kiesa ward (Mwanza Region)	2003 to 2004	Community, rural	Sero-prevalence survey	Outreach HTC at a central site	Laboratory testing, return for results in 2003/4 and POC testing in 2006/7	15 to 19 yrs	–	0.50	2244	223	9.9%	10.8%	9.1%	17	0.8%	7.6%	12.4%	2.0%	Moderate
Chamie *et al*. (2014)	Uganda	2012	Community, rural	Testing campaign	Outreach HTC	POC testing	10 to 19 yrs	–	–	1762	998	56.6%	–	–	5	0.30%	0.50%	–	–	Low
Bandason *et al*. (2013)	Zimbabwe, Harare	2010	Peri-urban	Schools	School-linked HTC	POC testing	5 to 9 yrs	–	0.54	2273	22	1.0%	1.0%	0.9%	1	0.04%	4.5%	7.6%	0.0%	Low
						10 to 11 yrs	–	0.56	1334	11	0.8%	0.8%	0.9%	1	0.1%	9.0%	16.6%	0.0%	Low

a HIV prevalence among all eligible;

b HIV prevalence among all tested. ANC, antenatal clinic; APC, acute primary care; HTC, HIV testing and counselling; IQR, interquartile ratio; PITC, provider-initiated testing and counselling; PHC, primary healthcare centre; VCT, voluntary counselling and testing; POC, point of care; N/A, not applicable.

### Uptake, yield and prevalence of HIV testing

There was great variability within a testing strategy. However, pooled proportions indicate that acceptance, yield and prevalence were highest when testing was offered in inpatient (86.3% [95% CI: 65.5 to 100], 12.2% [95% CI: 6.1 to 18.3], 15.4% [95% CI: 5.0 to 25.7]) and outpatient (69.5 [95% CI: 41.1 to 97.9], 7.4 [95% CI: 3.3 to 11.6], 11.3 [95% CI: 4.3 to 18.3]) settings as part of PITC in a range of settings (Table 2). Family-centred HTC mainly offered within PHCs had lower acceptance (51.7% [95% CI: 10.4 to 92.9]) yet higher yield (3.3% [95% CI: 1.7 to 4.9]) and prevalence (8.4% [95% CI: 3.4 to 13.5]) than home-based HTC (84.9% [95% CI: 74.4 to 95.4], 2.3% [95% CI: 0.7 to 4.0], 3.0% [95% CI: 1.0 to 4.9]). Outreach HTC strategies had the lowest acceptance (60.4% [95% CI: 23.4 to 97.4]), yield (0.6% [95% CI: 0.3 to 0.9]) and prevalence (1.3% [95% CI: 0.4 to 2.1]). One study from Zimbabwe which conducted HTC among primary school pupils at a nearby community centre reported extremely low acceptance (0.9%) and thus a very low yield (0.1%). This study was rated as low quality because of selection bias resulting from the use of a consecutive sample, from HTC being performed at specific times and from study outcome data not being stratified by age group (Supplementary file 4) [23].

**Table 2:** Ranges and summary estimates

	Acceptance rate (%)			Yield (%)			Prevalence (%)		
			
Strategy	Range	Pooled estimate (95% CI)	*I* ^2^, *p*	Range	Pooled estimate (95% CI)	*I* ^2^, *p*	Range	Pooled estimate (95% CI)	*I* ^2^, *p*
PITC, inpatient (*n*=4)	59.4 to 95.3	86.3 (65.5 to 100)	99.6, <0.01	4.7 to 23.3	12.2 (6.1 to 18.3)	91.1, <0.01	5.7 to 25.4	15.4 (5.0 to 25.7)	96.1, <0.01
PITC, outpatient (*n*=3)	40.7 to 96.5	69.5 (41.1 to 97.9)	99.8, <0.01	3.8 to 12.6	7.4 (3.3 to 11.6)	95.4, <0.01	5.3 to 15.9	11.3 (4.3 to 18.3)	96.0, <0.01
Family centred HTC (*n*=5)	9.8 to 100	51.7 (10.4 to 92.9)	100, <0.01	0.9 to 10.3	3.3 (1.7 to 4.9)	93.1, <0.01	2.3 to 18.1	8.4 (3.4 to 13.5)	98.9, <0.01
Home-based HTC (*n*=5)	56.5 to 99.4	84.9 (74.4 to 95.4)	99.8, <0.01	0.5 to 5	2.3 (0.7 to 4.0)	98.5, <0.01	0.5 to 5.6	3.0 (1.0 to 4.9)	98.6, <0.0s1
Outreach (*n*=5)	9.9 to 90.2	60.4 (23.4 to 97.4)	99.9, <0.01	0.3 to 2.1	0.6 (0.3 to 0.9)	55.5, 0.06	0.5 to 7.6	1.3 (0.4 to 2.1)	78.3, <0.01

CI, confidence interval; HTC, HIV testing and counselling; PITC, provider-initiated testing and counselling.

### Interpretations of findings

We investigated the uptake and yield of HTC among children and adolescents in SSA, the region where 90% of the world's HIV-infected children live [4]. One of the key findings of this review is the lack of evidence for HTC approaches that are targeted towards children and adolescents. The HTC strategies employed predominantly replicate strategies developed for adults, with little consideration of the specific barriers associated with HTC and the needs of this age group [20]. HIV test acceptance, yield and prevalence differed according to setting and strategy. Generally yield and prevalence are influenced by 1) the overall HIV prevalence in the target group; 2) the refusal rate and 3) whether or not refusal is associated with HIV risk. Yield and prevalence are similar when refusal rates are low. The yield takes into account the refusal rate and thus is appropriate for comparing different testing strategies. The prevalence determines the number needed to test in order to diagnose a new case of HIV and has cost and resource implications.

The most common HTC strategy was healthcare facility-based testing, which in general reported a high uptake of HTC, particularly in hospital inpatient settings. The HIV yield and prevalence were also high, underscoring the importance of implementing routine HTC in healthcare facilities in high HIV prevalence settings. Reported barriers to PITC include lack of clear guidelines around consent procedures, prioritization of HTC within PMTCT programmes over testing of children and adolescents and perceived lack of skills among healthcare providers to discuss HTC with children, adolescents and their guardians [20]. Despite the relatively high acceptance and yield of PITC as well as linkage to care (95%) [20], the crucial caveat is that this strategy mainly identifies children in inpatient settings when they are symptomatic and likely to have advanced disease [21, 22]. In contrast, community-based HTC approaches have the potential to diagnose children at an earlier stage of infection, as they do not rely on individuals presenting with symptoms. However, many studies reporting on such approaches tend to exclude children and adolescents [6].

Only one study, conducted in Zimbabwe, evaluated school-linked HTC among primary schoolchildren, which had the lowest acceptance rate compared to the other HTC strategies (1%) [23]. Key barriers described in this study were parents’ concern about confidentiality, stigma, inadvertent disclosure of their own HIV diagnosis and its likely adverse consequences. Healthcare workers were reluctant to test children (the majority of whom were orphaned) who had no legally-defined guardians, a concern which has also emerged in facility-based PITC [20]. South Africa is planning an extensive high school-linked HTC campaign, which forms part of the “basket of services” offered by the new Integrated School Health Program [3]. A recent qualitative study found that parents were generally in favour of school-linked HTC. However, they were not aware of their parental limitations in terms of the South African Children's Act, which acknowledges that consent for an HIV test may be given by the child, if the child is over 12 years of age [29]. The inability to consent to HTC due to legal age restrictions in other countries in the region poses a challenge to school-linked testing programmes. This situation poses an additional barrier to HTC in Africa, where minors often live in extended families with no clearly defined guardian, as parents may have died or be absent for work [30–32]. School-linked HTC warrants further rigorous investigation into the appropriate age group to target, age at which one can provide consent and methods of subsequent referral to HIV care, given the high HIV prevalence reported in a South African survey among high school learners (4.7%), particularly among teenage girls (7.7%) [33].

Four studies reported on family-centred testing whereby an adult patient on ART (the parent) acts as the index case and triggers testing of the whole household, including children and adolescents at risk [15–17]. This method of case finding has been employed in the context of tuberculosis contact tracing for decades [34, 35]. The vast majority of HIV infections in SSA are acquired sexually and vertically, resulting in strong spatial clustering of HIV within households [36]. Family-centred HTC was either offered to invited individuals at PHC [15, 16, 19] or through home-based HTC [17, 19], which might explain the difference in uptake. The Kenyan family-centred model reported a relatively low acceptance rate (32.5 to 57.0%), but a high prevalence among adolescents tested (11.4 to 18.1%). The former might be due to logistical problems such as transport, a challenge often reported in the context of tuberculosis contact tracing [37–39]. This could be addressed by testing family members in the household rather than making them come to the healthcare facility. Home-based family-centred HTC resulted in a six times increase in test uptake among 6- to 14-year-olds in Uganda compared to family-centred HTC provided at PHCs [19].

Outreach HTC strategies had a low acceptance, yield and prevalence across all HTC strategies. The wide range of acceptance rates among studies investigating outreach HTC might be explained by differences in testing methods (POC vs. laboratory based testing) and differences in denominators. The study by Chamie *et al*. [11] was conducted as part of a testing campaign and denominators were estimated from a previous household census. Thus individuals not at home during the testing campaign were counted as eligible, resulting in an underestimation of test acceptance. Mobile HTC and outreach strategies have been successfully implemented in many SSA settings [40–42]. However, only one study included in this review used an outreach approach in the context of a testing campaign [11]. All other studies using outreach or mobile services were conducted as part of community prevalence surveys and as such were not representative. However, a recent cluster randomized trial investigating the effect of mobile community-based HTC resulted in a significant increase in testing rates among 16- to 17-year-olds [43].

### Strength and limitations

This review has several strength and limitations. We used an extensive search strategy including multiple databases and conference abstracts without language restrictions. Anticipating that data on adolescents might be reported as part of paediatric and adult studies, we included adult and paediatric studies in our search strategy and contacted authors of potentially eligible studies to obtain data disaggregated by age. However, additional data was only obtained for one-fifth of potentially eligible studies. As with any systematic review, this review is subject to publication bias. Specifically testing strategies with low acceptability might be less likely to be published in peer-reviewed literature. Due to paucity of data, this review was unable to assess the differences in outcomes disaggregated by age and whether HIV was acquired vertically (i.e. perinatally infected long-term survivors) or horizontally. Six of fourteen studies reported a similar HIV prevalence in girls compared to boys, which might indicate that those studies mainly targeted vertically infected children. Finally, none of the included studies assessed linkage to care, cost or cost-effectiveness, which are important factors policy makers need to consider when deciding which strategies to implement.

## Conclusions

Achieving universal coverage of HTC for key populations in SSA under scarce resource constraints will require the implementation of innovative, effective and economically efficient population-based HTC strategies which can be readily brought to scale [4]. Data from our review indicate that HTC approaches delivered within communities outside of a healthcare facility (i.e. home-based, family-centred and outreach) have a high acceptance among this priority age group. Additionally these strategies have the potential to identify individuals early in their stage of HIV infection. However, there is a paucity of data on HTC strategies that extend beyond the healthcare facility for children and adolescents, particularly in areas where data are scarce such as school-linked, family-centred and mobile HTC. Moreover it is necessary to assess linkage to care and the cost-effectiveness of these different HTC approaches. Thus further evaluations are required prior to policy makers and programme managers planning for their scale-up. Furthermore, qualitative studies establishing the barriers to testing for this age group should be encouraged. Those barriers are likely to be specific to both the testing strategy and the setting [20, 44].

## Supplementary Material

Uptake and yield of HIV testing and counselling among children and adolescents in sub-Saharan Africa: a systematic review

Uptake and yield of HIV testing and counselling among children and adolescents in sub-Saharan Africa: a systematic review

Uptake and yield of HIV testing and counselling among children and adolescents in sub-Saharan Africa: a systematic review

Uptake and yield of HIV testing and counselling among children and adolescents in sub-Saharan Africa: a systematic review
