# Supplementary material for: Uptake and yield of HIV testing and counselling among children and adolescents in sub-Saharan Africa: a systematic review
Source: J Int AIDS Soc. 2015 Oct 14;18(1):20182. doi: 10.7448/IAS.18.1.20182 (PMC4607700; doi:10.7448/IAS.18.1.20182)
Supplement: Uptake and yield of HIV testing and counselling among children and adolescents in sub-Saharan Africa: a systematic review [file JIAS-18-20182-s001.pdf]

HIV Testing and Counselling Strategies among Children & Adolescents in sub-Saharan Africa

Protocol

1) AIMS

To assess the acceptance rate, yield and prevalence of different HIV testing and counselling (HTC) strategies among children and adolescents in a variety of settings.

2) METHODS

2.1) Criteria for considering studies for this review

a) Types of studies

- Randomised and non-randomised trials
- Prospective and retrospective cohort studies
- Cross-sectional studies
- Programme evaluation

b) Types of participants

Children and adolescents aged 5 – 19 years with HIV status unknown.

c) Type of intervention

Any method of HIV testing strategy (e.g. home based testing, provider initiated counselling and testing and school based testing).

d) Eligibility criteria

To be included in the review, an article has to meet the following inclusion criteria:

1. Describe an HIV testing strategy with information required to generate the acceptability, yield and prevalence of the strategy (I.e. Data regarding: (1) The total number of participants to whom the testing strategy was offered; (2) The total number of participants who accepted testing and received the result; and (3) The total number of participants who tested positive).

2. The participants tested for HIV need to be aged 5-19 years. Studies where the age group does not overlap by at least 3 years will be excluded.
3. The study was conducted in Sub-Saharan Africa and published after 2003
4. No restrictions are placed based on language.

Exclusion criteria:

Anonymous sero-prevalence surveys will be excluded. Studies conducted in antenatal settings as part of the prevention of mother to child transmission, inpatients, outpatients, STI clinics and TB clinics will only be included for full text review if the abstract clearly indicates that the age-range includes at least 3 years overlapping the age-range of 5-19 years. Studies with unclear denominators will be excluded from the review.

3.2) Types of outcome measures

a) Primary outcomes

- *Acceptance rate:* the proportion of individuals who underwent HTC and received their test results of those eligible for HTC
- *Yield of new HIV-positive diagnosis:* the proportion of individuals who were newly-diagnosed HIV-positive of those who were eligible for HTC
- *Prevalence of new HIV-positive diagnosis:* the proportion of individuals who were newly-diagnosed HIV-positive of those who underwent HTC.

3.3) Search methods

a) Electronic searches

We will develop a compound search strategy to identify all relevant studies regardless of language or publication status. We will limit the search to sub-Saharn Africa and publications occurring between 1 January 2004 and 30 September 2014. We will search the following electronic databases:

- MEDLINE
- EMBASE
- Global Health

b) Other resources

- Reference lists: We will check the reference lists of all studies identified by the above methods and examine bibliographies of systematic reviews or meta-analyses[1, 2].

- Conference abstracts: We will search conferences, where abstract databases are available online [i.e. Conference on HIV Pathogenesis and Treatment of the International AIDS Society (IAS, 2005 - 2011), the Conference on Retroviruses and Opportunistic Infections (CROI, 2011-2013) and the International AIDS Conference (AIDS, 2004 - 2012).
- Authors of eligible and potentially eligible studies: We will contact these authors for age-stratified data if applicable; at least two attempts will be made to contact the author.

3.4) Data collection and analyses

a) Selection of studies

All references identified by the compound search strategy will be imported into EndNote. Duplicates will be removed. Titles and abstracts will be examined by DG and KK working independently. The full text will be obtained for potentially relevant studies and the inclusion criteria will be applied. The inclusion criteria will be applied by both authors independently using a standardised eligibility form.

b) Data extraction and management

After initial search and article screening, DG and KK will independently double-code and enter information from each selected study onto standardised data extraction forms. Extracted information will include:

Study details: citation, start and end dates, location, study design and details.

Participant details: study population, age range and gender

Intervention details: detailed description of the type of testing strategy

Outcome details: Total number of participants who were offered HIV testing, total number of participants that accepted testing and received their results, and the total number of participants that tested positive.

c) Assessment of risk of bias in included studies

Both authors will independently assess the risk of bias. Studies will be categorised into 3 groups depending on the level of bias: low risk of bias, high risk of bias or unclear risk of bias.

The following will be assessed:

1. Was the HIV testing strategy clearly described?
2. Were the HIV test results for each individual confirmed?
3. Are the study results generalisable to this method of HIV testing?
4. Was there any selection bias?

d) Analysis

Proportions and corresponding 95% confidence intervals will be presented for HTC acceptance, yield and HIV prevalence. Data for each outcome will then be pooled and stratified by testing strategy. Pooled proportions and 95% confidence intervals will be estimated using a random effects model, weighting for the inverse of the variance. Data analyses were conducted using Stata 12 (Statacorp, College Station, Tx).

4) TIMETABLE

Milestones over the next three months of 2013 are given in the table below.

	**July**	**August**	**September**
Development of search strategy			
Contacting of experts in the field, 1st attempt			
Contacting of experts in the field, 2nd attempt			
Search of conference abstracts			
Screening of titles and abstracts			
Screening of full text papers			
Final inclusion of studies			
Data extraction and entry			
Data synthesis and analyses			
Quality assessment			
Writing up of report			

5) REFERENCES

1. Sabapathy K, Van den Bergh R, Fidler S, Hayes R, Ford N. Uptake of home-based voluntary HIV testing in sub-Saharan Africa: a systematic review and meta-analysis. PLoS Medicine / Public Library of Science. 2012;9(12):e1001351. PubMed PMID: 23226107. English.
2. Kennedy C, Fonner V, Sweat M, Okero FA, Baggaley R, O'Reilly K. Provider-Initiated HIV Testing and Counseling in Low- and Middle-Income Countries: A Systematic Review. AIDS Behav. 2013 2013/06/01;17(5):1571-90. English.
