# Supplementary material for: Uptake and yield of HIV testing and counselling among children and adolescents in sub-Saharan Africa: a systematic review
Source: J Int AIDS Soc. 2015 Oct 14;18(1):20182. doi: 10.7448/IAS.18.1.20182 (PMC4607700; doi:10.7448/IAS.18.1.20182)
Supplement: Uptake and yield of HIV testing and counselling among children and adolescents in sub-Saharan Africa: a systematic review [file JIAS-18-20182-s003.pdf]

Appendix 3: Search strategy

SET	TOPIC	SEARCH TERMS
1	Adolescents	ADOLESCENT or adolescent
2		CHILD or child or children
3		YOUNG ADULT or young adj1 adult
4		Youth
5		PEDIATRICS or paediatric
6	Set 1-5 were combined with "or"	
7	HIV	AIDS SERODIAGNOSIS
8		HIV SEROPOSITIVITY
9		HIV or hiv
10	Set 7-9 were combined with "or"	
11	Testing	provider adj1 initiated adj1 counselling adj1 and adj1 testing
12		pitc
13		home adj1 based adj1 testing
14		community adj1 based adj1 testing
15		voluntary adj1 counselling adj1 testing
16		vct
17		Pmtc
18		school adj1 based adj1 testing
19		hiv adj1 test
20		hiv adj1 testing
21		routine adj1 testing
22		opt adj1 out
23		antenatal adj1 testing
24	Set 11-23 were combine with “or”	
25	Set 6, 10, 24 were combined with “and”	

The search was restricted to sub-Saharan African countries and publications published after 2003.
