# Supplementary material for: Uptake and yield of HIV testing and counselling among children and adolescents in sub-Saharan Africa: a systematic review
Source: J Int AIDS Soc. 2015 Oct 14;18(1):20182. doi: 10.7448/IAS.18.1.20182 (PMC4607700; doi:10.7448/IAS.18.1.20182)
Supplement: Uptake and yield of HIV testing and counselling among children and adolescents in sub-Saharan Africa: a systematic review [file JIAS-18-20182-s004.pdf]

## Appendix 4: Quality assessment

| | | | Testing procedures | Selection | Interpretation of findings | | | | | |
|---|---|---|---|---|---|---|---|---|---|---|
| **Author, Year** | **Context** | **HTC strategy** | **Clear description of HTC algorithms and validation of results** *Maximum score=2* | **Good representativeness of the sample** *Maximum score=2* | **Clear description of HTC outcome data** *Maximum score=2* | **Major limitations discussed** Maximum score=2 | **Important sub-group analyses performed (i.e. HTC data stratified by age-group and sex)** *Maximum score=2* | **Total score** | **Overall quality:** *High: Total score= 8-10; Moderate: Total score= 5-7; Low: Total score= 0-4* | **Main comments** |
| Ferrand (2010)[21] | Inpatient | PITC | 2 | 1 | 1 | 2 | 0 | 6 | Moderate | Recruitment conducted on weekdays only |
| Wanyenze (2010)[18] | Inpatient | PITC | 1 | 1 | 1 | 1 | 1 | 5 | Moderate | Analysed routine hospital records with moderate levels of missing data |
| Abbas (2010)[27] | Inpatient | PITC | 1 | 0.5 | 1 | 0 | 0 | 2.5 | Low | Consecutive sample drawn. Limitations not discussed. Testing procedures, participant flow and handling of known HIV-positives in the analysis are unclear |
| Kankasa (2009)[28] | Inpatient | PITC | 1 | 1 | 0.5 | 0.5 | 1 | 4 | Low | Consecutive sample drawn. Limitations of the study design and methods not sufficiently discussed. HTC outcome data not disaggregated by age |

| | | | | | | | | | | |
|---|---|---|---|---|---|---|---|---|---|---|
| Ramirez-Avila (2012)[25] | Outpatient | PITC | 2 | 0.5 | 2 | 1 | 0.5 | 6 | Moderate | Analysed routine records from a semi-private facility, hence data may not be generalisable to public sector clinics. Missing data not described. |
| Kranzer (2014)[31] | Outpatient | PITC | 2 | 1.5 | 1 | 1 | 1 | 6.5 | Moderate | High proportion of missing data for children who refused HTC |
| Ferrand (2010)[22] | Outpatient | PITC (n=506), ANC (n=88) | 2 | 1 | 1 | 1 | 1 | 6 | Moderate | Consecutive sample drawn. HTC outcome data not stratified by age |
| Mongare (2013)[15] | Outpatient | Family-centred HTC | N/A | N/A | N/A | N/A | N/A | N/A | N/A | Abstract- quality assessment not done |
| Kulzer (2012)[16] | Outpatient | Family-centred HTC | 2 | 0.5 | 1 | 1 | 0 | 4.5 | Low | Routine facility records used. Baseline data of sample and proportion of missing data not provided |
| Were (2006)[17] | Home-based | Family-centred HTC | 2 | 0.5 | 1 | 0 | 1 | 4.5 | Low | Consecutive sample drawn. Limitations not discussed |
| Lugada (2010)[19] | Home-based | Family-centred HTC | 2 | 1 | 1 | 1 | 1 | 6 | Moderate | Sub-study of a cluster-randomised trial. Households visited at specific times. HTC outcome data-Adolescents grouped with younger adults |
| Lugada (2010)[19] | Outpatients | Family-centred HTC | 2 | 1 | 1 | 1 | 1 | 6 | Moderate | Sub-study of a cluster-randomised trial. Households visited at specific times. Adolescents grouped with younger adults |

| | | | | | | | | | | |
|---|---|---|---|---|---|---|---|---|---|---|
| Naik (2012)[24] | Testing campaign | Home-based HTC | 2 | 2 | 1 | 1 | 1 | 7 | Moderate | HTC outcome data-Adolescents grouped with younger adults |
| Wachira (2014)[29] | Testing campaign | Home-based HTC | 0.5 | 0.5 | 0.5 | 0.5 | 1 | 3 | Low | Consecutive sample drawn. Retrospective record review of routine records. HTC outcome data not disaggregated by age |
| Vreeman (2010)[13] | Testing campaign | Home-based HTC | 2 | 0.5 | 2 | 1 | 0.5 | 6 | Moderate | HTC was not universally offered to children in the household (eligibility for HTC was based on characteristics of the mother) |
| Dalal (2013)[14] | Sero-prevalence survey | Home-based HTC | 2 | 0.5 | 2 | 1 | 1 | 6.5 | Moderate | Sampling strategy is unknown. Study is nested within a surveillance programme |
| Angotti (2009)[26] | Sero-prevalence survey | Home-based HTC | 1.5 | 1 | 1 | 1 | 0.5 | 5 | Moderate | HTC outcome data not stratified by age |
| Kranzer (2011)[8] | Sero-prevalence survey | Outreach (Mobile clinic with home-based invitation) | 2 | 1 | 1 | 1 | 0.5 | 5.5 | Moderate | HTC outcome data not stratified by age |
| Baisley (2012)[9] | Sero-prevalence survey | Outreach HTC at central site (opt out) | 2 | 1 | 1 | 1 | 0.5 | 5.5 | Moderate | HTC outcome data-Adolescents grouped with younger adults |
| Baisley (2012)[9] | Sero-prevalence survey | Outreach HTC at central site (opt in) | 2 | 1 | 1 | 1 | 0.5 | 5.5 | Moderate | HTC outcome data-Adolescents grouped with younger adults |

| | | | | | | | | | | |
|---|---|---|---|---|---|---|---|---|---|---|
| Isingo (2012)[10] | Sero-prevalence survey | Outreach HTC at a central site | 2 | 2 | 1 | 1 | 0.5 | 6.5 | Moderate | HTC outcome data-Adolescents grouped with younger adults and data not stratified by age |
| Chamie (2014)[11] | Testing campaign | Outreach HTC | 1 | 0.5 | 0.5 | 1 | 0.5 | 3.5 | Low | HTC performed mainly on weekdays, with lower uptake among younger individuals. Individuals not at home were counted as eligible. HTC outcome data not disaggregated by age |
| Bandason (2013)[23] | Schools and community centres | School-linked HTC | 2 | 0.5 | 0.5 | 1 | 0.5 | 4.5 | Low | Consecutive sample drawn, HTC performed at select times during the day. HTC outcome data not stratified by age |
